# Optimised Sunflower Oil Content for Encapsulation by Vibrating Technology as a Rejuvenating Solution for Asphalt Self-Healing

**DOI:** 10.3390/polym15061578

**Published:** 2023-03-22

**Authors:** Jose L. Concha, Rodrigo Delgadillo, Luis E. Arteaga-Pérez, Cristina Segura, Jose Norambuena-Contreras

**Affiliations:** 1 LabMAT, Department of Civil and Environmental Engineering, University of Bío-Bío, Concepción 4051381, Chile; 2 Departamento de Obras Civiles, Universidad Técnica Federico Santa María, Valparaíso 2390123, Chile; 3 LPTC, Department of Wood Engineering, University of Bío-Bío, Concepción 4051381, Chile; 4 Unidad de Desarrollo Tecnológico, Universidad de Concepción, Coronel 4191996, Chile

**Keywords:** asphalt self-healing, sunflower oil, rheology, bitumen rejuvenation, capsule dosage

## Abstract

This study aimed to determine an optimal dosage of sunflower oil (i.e., Virgin Cooking Oil, VCO) as a rejuvenator for asphalt self-healing purposes, evaluating its effect on the chemical (carbonyl, and sulfoxide functional groups), physical (penetration, softening point, and viscosity), and rheological (dynamic shear modulus, and phase angle) properties of long-term aged (LTA) bitumen. Five concentrations of sunflower oil (VCO) were used: 1%, 2%, 3%, 4%, and 5% vol. of LTA bitumen. VCO was encapsulated in alginate biopolymer under vibrating jet technology using three biopolymer:oil (B:O) mass ratios: 1:1, 1:5, and 1:9. The physical, thermal, and mechanical properties of the capsules were studied, as well as their effect on the physical properties of dense asphalt mixtures. The main results showed that an optimal VCO content of 4% vol. restored the chemical, physical, and rheological properties of LTA bitumen to a short-term ageing (STA) level. VCO capsules with B:O ratios of 1:5 presented good thermal and mechanical stability, with high encapsulation efficiency. Depending on the B:O ratio, the VCO capsule dosage to rejuvenate LTA bitumen and asphalt mixtures varied between 5.03–15.3% wt. and 0.24–0.74% wt., respectively. Finally, the capsule morphology significantly influenced the bulk density of the asphalt mixtures.

## 1. Introduction

During the last years, a great interest has emerged in the research of multifunctional asphalt pavement structures incorporating self-healing ability by encapsulated rejuvenators [1,2]. This technology acts on the binder element of the asphalt mixture, i.e., the bitumen, a viscoelastic material with natural self-healing ability [3,4]. With in-service lifetime, the bitumen loses its viscoelasticity due to the ageing caused by long-term exposure to light, heat, oxygen, and traffic load [5], thus, the self-healing capacity of bitumen gradually decreases [3]. As a result, ageing of bitumen leads to changes in its saturates, aromatics, resins, and asphaltenes (SARA) molecular fractions, increasing the net asphaltene content [6,7]. This modifies the chemical balance of the carbonyl and sulfoxide functional groups [8], promoting the increase in molecular polarity and agglomeration potential between bitumen molecules [9]. Such changes have an impact on the physical and rheological properties of the bitumen, increasing its softening point, viscosity, and complex shear modulus |G*|, while decreasing its penetration and phase angle (δ) [10,11,12]. This results in a more brittle bitumen, so microcracks appear and propagate on the pavement [13,14].

Encapsulated rejuvenators (i.e., capsules) are used to revert the impact of ageing on the physical and rheological properties of the bitumen. The encapsulated oils promote a rejuvenation based on the softening of the aged bitumen in all the pavement layers [2], restoring the (physical) asphaltenes/maltenes ratio of the aged bitumen [15,16]. Its working principle is based on the capsules being activated when a microcrack starts to propagate inside the aged bituminous material next to them, initiating a rheological rejuvenation process. The capsules will break or deform inside the asphalt mixture, releasing the healing agent—an oil with low viscosity and a high proportion of aromatic components [17]. The rejuvenator diffuses into the asphalt matrix and softens the aged bitumen, allowing the rejuvenated bitumen to flow through the open microcracks, thus, facilitating the crack’s self-healing to extend the life-span of the pavement infrastructure [18].

**Figure 1:**
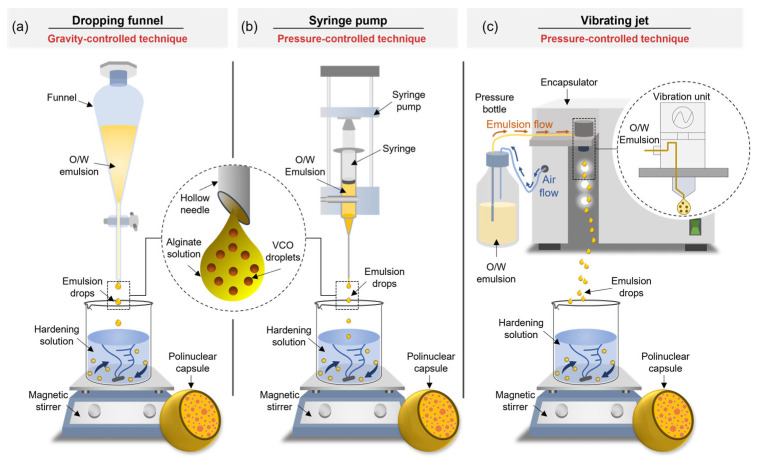
Illustration of (**a**) dropping funnel, (**b**) syringe pump, and (**c**) vibrating jet (nozzle) techniques to synthesise alginate-based capsules with a polynuclear microstructure.

Nowadays, capsules with polynuclear morphology have been extensively developed for asphalt self-healing purposes [18,19,20,21,22,23,24]. This type of capsule has a spherical shape with internal cavities or pores where the oil is enclosed [18,20], providing a controlled release of the restoring agent once the capsule is activated allowing multiple healings on aged bitumen over time [19]. The synthesis of polynuclear capsules can be achieved by extrusion-based methods employing dropping funnel [20,21,22] and syringe pump [20,22,23] devices. However, these methods have a drawback of lower production rates. Such a limitation can be surpassed by implementing more efficient encapsulation processes such as the vibrating jet (nozzle) technique, with the ability to produce a continuous flow of drops/capsules instead of the discrete flow obtained by dropping funnel or syringe pump (Figure 1) [25]. This method is based on the principle of a laminar jet break-up by the application of a vibrational frequency with defined amplitude to the extruded jet [2].

As encapsulating material, the alginate biopolymer has become of great interest [26] due to (i) its low thermal degradation at the temperature conditions of asphalt mixing; and (ii) its good mechanical stability to potentially resist the compaction pressure during paving operation [19]. Regarding the use of rejuvenators, the National Center for Asphalt Technology (NCAT) [27] and the Belgian Road Research Centre (BRRC) [28] includes a common category of rejuvenator coming from vegetable sources including triglycerides and fatty acids and vegetable oil from agroindustry, respectively. Based on this, several studies [19,29,30] have proposed the sunflower oil (named virgin cooking oil—VCO in this study) as a promising rejuvenator with softening effect for long-term aged (LTA) bitumen. The work of Shirzad et al. [29] found that adding 5% wt. of VCO into bitumen improved its rheological properties, reversing the stiffening of short-term aged (STA) and long-term aged (LTA) bitumen compared to a commercial rejuvenator. Similarly, Tarar et al. [30] evidenced a reduction in |G*| when VCO was added in a content of 5% wt. of bitumen, improving its fatigue performance.

About the encapsulation of VCO in alginate-based matrices, Ruiz-Riancho et al. [23] proved that polynuclear VCO-alginate capsules were thermally stable during the mixing and compaction processes, and that they release between 10% and 46% of their total payload under compression load. In addition, Norambuena-Contreras et al. [31] concluded in 2019 that adding 0.5% wt. of VCO capsules enhanced the indirect tensile strength (ITS) and fatigue life of a dense asphalt mixture, while optimising the self-healing of damaged asphalt samples without affecting their rheological properties. Wang et al. [32] confirmed a good distribution of VCO capsules for contents of 0.5% wt. of warm- and hot-mix asphalt. The capsules released about a 5.8% wt. and a 10.4% wt. of encapsulated VCO for each mixture, respectively, during transportation and compaction simulation.

**Figure 2:**
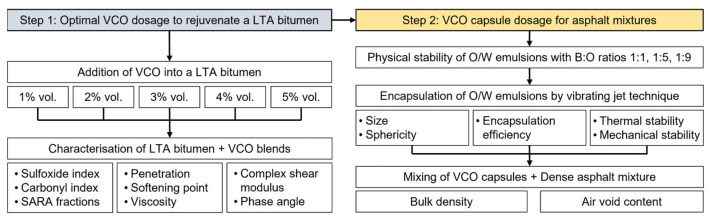
Flowchart of the experimental study.

Although the promissory results on the use of VCO and its encapsulation to recover the physical and rheological properties of long-term aged asphalt, there remains a research gap in proposing a dosage method of VCO capsules that relies on the rejuvenating effect of the pure oil on LTA bitumen. This method should select a capsule dose based on the chemical, physical, and rheological rejuvenation effects of the VCO on the LTA bitumen. A sub-estimated dosage of the capsules could not meet a proper rejuvenation target of an LTA bitumen. On the contrary, an over-estimated dosage of the capsules will over-rejuvenate the aged bitumen, so the asphalt mixture is prone to permanent deformations. As starting point, Xu et al. [33] stated that encapsulated rejuvenators should not over-rejuvenate the bitumen to a grade lower than its grade after paving (RTFO condition). Given this rejuvenation target, some research questions emerge: (1) how can experimental testing be utilised to determine the optimal amount of VCO required for rejuvenating an LTA bitumen? and (2) how can the appropriate dosage of VCO capsules be determined to achieve a rejuvenating effect equivalent to that of pure VCO when added to LTA bituminous materials? To respond to the previous questions, the main objectives of this work were to (1) evaluate the effect of the VCO content as a rejuvenator on the chemical, physical, and rheological properties of LTA bitumen to determine its proper dosage and (2) encapsulate the VCO as a rejuvenator by vibrating jet technology for asphalt self-healing purposes. Experimental tests were carried out to characterise the rejuvenating effect of VCO in contents between 1–5% by vol. of LTA bitumen and determine its proper dosage as encapsulated additive for bitumen and asphalt mixtures; see detail in Figure 2.

## 2. Materials and Methods

### 2.1. Materials

Polynuclear VCO capsules were synthesised. The polymeric encapsulating matrix of the capsules consisted of low-viscosity sodium alginate powder (Mannuronic/Guluronic ratio of 0.92, density 1.02 g/cm^3^, viscosity ≤ 300 mPa∙s in a 2% wt. solution @ 20 °C), provided by Buchi (Flawil, Switzerland) and calcium-chloride dihydrate (CaCl_2_·2H_2_O) at 77% purity, provided by Winkler (Concepción, Chile). On the other hand, virgin cooking oil (VCO) from commercial sunflower oil (density 0.85 g/cm^3^, viscosity 70 mPa∙s @20 °C, pH 5.3–5.5) was encapsulated as a bitumen rejuvenator. Semi-dense asphalt mixtures with and without VCO capsules were also used in this study, consisting of aggregates in sizes from 0.075 to 12.5 mm (see the granulometry used in Table 1), and virgin bitumen CA-24 (penetration grade 50/70, softening point 52.2 °C, and density 1.034 g/cm^3^ @ 20 °C) added in a content of 5% wt. of the total mass of aggregates. Both bitumen and aggregates were provided from a local pavement company located in Concepción, Chile.

The virgin bitumen was aged under the rolling thin film oven (RTFO) and the pressure air vessel (PAV) laboratory processes, for STA and LTA, respectively. The RTFO test was conducted in a quality control laboratory of a local bitumen company from Concepción, Chile, according to ASTM D 2872-19 [34]. The bitumen is kept at 163 °C during 75 min, with forced air circulation, simulating the ageing that happens during plant mixing, transportation, and paving (STA). The PAV test was carried out under the ASTM D 6521-19 [35]. The bitumen already RTFO-aged is subjected to 2.070 kPa of pressure at 100 °C during 20 h, simulating the bitumen’s ageing after 5–7 years of service life (LTA). In the following sections, the STA bitumen will be referred as RTFO and the LTA bitumen as PAV, for more clarity about the ageing procedures applied.

### 2.2. Blending Process of the PAV Bitumen with VCO

Five PAV-aged bitumen blends containing VCO in concentrations varying from 1% to 5% vol. bitumen were produced in this study. First, 300 mL of PAV bitumen was poured into a 950 mL (1/4 gallon) paint can and heated at 140 °C in a heating plate, being mechanically stirred (REMI, model RQG 126/D, Mumbai, India) at 300 rpm for 15 min. While stirring the PAV-aged bitumen, the VCO dosage was added dropwise using a pipette. The PAV bitumen samples were then cooled to ambient temperature and hermetically sealed for further chemical, physical, and rheological analyses.

### 2.3. Chemical Characterisation of the Bitumen Samples

The virgin RTFO and PAV bitumen with and without VCO were chemically characterised by Fourier transformed infrared (FT-IR) spectroscopy coupled with attenuated total reflectance (ATR) mode and the quantification of the saturates, aromatics, resins, and asphaltenes (SARA) fractions. FTIR-ATR tests were recorded over 32 scans in the range of 4000 cm^−1^ to 600 cm^−1^, with a resolution of 4 cm^−1^ using a Nicolet iS20 spectrometer equipped with a DTGS detector and CaF_2_ windows (Thermo Fisher Scientific, Waltham, MA, USA) and an ATR accessory (Quest Specac, Orpington, UK). The signal processing consisted of baseline, ATR correction for diamond crystal, and normalisation as proposed by Hofko et al. [36]. The chemical changes associated to the oxidation of the virgin bitumen and the rejuvenation of the PAV-VCO bitumen samples were estimated by the carbonyl ($C_{I}$), sulfoxide ($S_{I}$), and rejuvenator ($R_{I}$) indices determined as the proportion between the peak areas of the carbonyl (1700 cm^−1^) and sulfoxide (1030 cm^−1^) functional groups and the rejuvenator peak (1743 cm^−1^) and a reference area ($\Sigma_{RA}$), respectively—see Equations (1)–(4):

(1) $$C_{I}=\frac{A_{1700}}{\Sigma_{RA}}$$

(2) $$S_{I}=\frac{A_{1030}}{\Sigma_{RA}}$$

(3) $$R_{I}=\frac{A_{1743}}{\Sigma_{RA}}$$

(4) $$\Sigma_{RA}=A_{\left(2953,\;2862\right)}+A_{\left(1700\right)}+A_{\left(1600\right)}+A_{\left(1460\right)}+A_{\left(1376\right)}+A_{\left(1030\right)}+A_{\left(864\right)}+A_{\left(814\right)}+A_{\left(743\right)}+A_{\left(724\right)}$$

Additionally, a combined index was determined as the sum of $C_{I}$ and $S_{I}$ indices. The SARA fractions were determined under a modification of the standard ASTM D 4124-09 [37] using a two-step separation process as detailed described by Concha et al. [19]. The first step consisted of the separation of the maltenic fraction from the asphaltenes using a reflux apparatus and n-Heptane. A second separation of the maltenic components, i.e., saturates, aromatics, and resins, was carried out by column chromatography using solutions of n-Heptane, methanol and methanol:toluene, respectively. All the reagents were acquired from Merck (Darmstadt, Germany).

### 2.4. Physical Characterisation of the Bitumen Samples

The physical properties of the bitumen samples were characterised by (i) penetration under ASTM D 5-97 [38], using a Humboldt H-1240 penetrometer (Elgin, IL, USA); (ii) softening point under ASTM D 36-06 [39], using a Humboldt H-1569 ring-and-ball apparatus (Elgin, IL, USA); and (iii) dynamic viscosity tests under ASTM D 4402-06 [40] measured in a range of temperature from 60 to 160 °C, at 10 °C increments using a RVDV-II+Pro rotational viscometer (Brookfield, Middleboro, MA, USA).

### 2.5. Rheological Characterisation of the Bitumen Samples

The rheological response of each bitumen sample was characterised using a dynamic shear rheometer Physica MCR 301 and the software Anton Paar RheoCompass (Anton Paar, Graz, Austria), under ASTM D7175-15 [41]. Frequency and temperature sweep tests were performed at a constant strain amplitude of 0.5%, a range of frequencies 0.1–100 rad/s, and a range of temperatures from 5 to 75 °C, at 5 °C increments, using 8 mm and 25 mm plates. Based on the time–temperature superposition principle detailed explained in Papagiannakis and Masad [42], the master curves of dynamic shear modulus (|G*|) and phase angle (δ) of the bitumen samples were obtained at a reference temperature of 25 °C.

### 2.6. Synthesis and Characterisation of the VCO Capsules and Their Components

**Figure 3:**
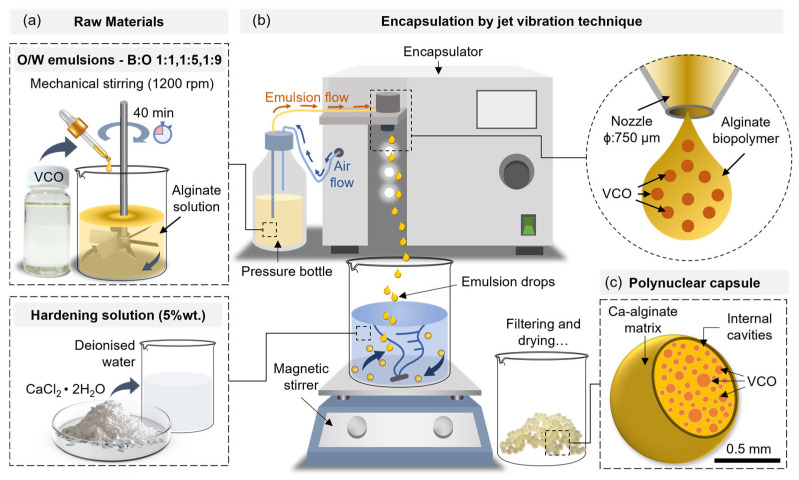
Synthesis of the capsules by vibration (nozzle) technology. (**a**) Materials used for encapsulation; (**b**) encapsulation of the O/W emulsions; and (**c**) polynuclear structure of the capsules.

Polynuclear VCO capsules were synthesised by a vibrating jet method based on the ionic gelation principle of alginate and the Ca^2+^ ions from the CaCl_2_·2H_2_O. The process consisted of two steps, as follows:

(1) The first step consisted of the synthesis of oil-in-water (O/W) emulsions. For this, a 2% wt. sodium alginate solution was prepared by using a magnetic stirrer (SCI550-S, Model OS40-Pro-LB Pro, Rocky Hill, CO, USA) at 250 rpm for 24 h. After this, the alginate solution was mechanically agitated at 1200 rpm for 40 min while the VCO was incorporated dropwise at three biopolymer:oil mass (B:O) ratios; 1:1, 1:5, and 1:9, resulting in three O/W emulsions with nominal viscosities @20 °C of 200.4 mPa·s, 241.50 mPa·s, and 315.5 mPa·s, respectively. The physical stability of the emulsion was evaluated by the creaming index. For this, a volume of 30 mL of each emulsion was poured into a glass vial and maintained in response for a period of 30 h. During this time, the degree of separation of the continuous (alginate solution) and disperse (VCO droplets) phases of the emulsion was measured at 0 h, 1 h, 2 h, 3 h, 6 h, 12 h, 24 h, 26 h, 28 h, and 30 h. At each time, it was determined the creaming index for all the emulsions according to the method described in Concha et al. [19] and Norambuena-Contreras et al. [18]. At each of the previous times, the size of the VCO droplets in the O/W emulsion were measured by fluorescence microscopy images (ICOE IV 5100FL, Ningbo, China) processed in the ImageJ^®^ software (Fiji distribution, version 1.52p, National Institutes of Health, Bethesda, MD, USA).
(2) A second step consisted of the encapsulation of each of the O/W emulsions by means of the vibrating jet technique using a Buchi encapsulator (B-390, Flawil, Switzerland). For this, each emulsion was pumped by applying 450–550 mbar of air pressure through a nozzle of 750 µm coupled to a vibrating unit settled to 350 Hz. With these settings, the outgoing O/W emulsion with a laminar flow from the nozzle was: (i) broken up by vibration, (ii) separated into droplets, and (iii) collected in a 5% wt. CaCl_2_∙2H_2_O hardening solution agitated at 250 rpm using a magnetic stirrer (see Figure 3). To prevent the droplets from hitting each other in flight, an electro-static charge of 1500 V was applied on the surface of the outgoing emulsion droplets from the nozzle. The freshly prepared capsules were filtered from the hardening solution and rinsed with 250 mL of deionised water later dried in an oven at 30 °C for 24 h. This resulted in the synthesis of three VCO capsule designs varying their B:O ratios, identified as 1:1, 1:5, and 1:9. Finally, the VCO capsules were stored in a freezer at -10 °C, preventing the VCO from excessive oxidation.

The morphology of the polynuclear capsules was characterised by their size, using an optical microscope (Leica EZ4, Wetzlar, Germany) with 35× magnification. The size was statistically evaluated as the diameter of 100 randomly selected capsules using the software ImageJ^®^. The sphericity factor (SF) of the capsules was determined based on the measurement of the maximum diameter of the capsule and its perpendicular projection as proposed by Alpizar-Reyes et al. [43]. The encapsulation efficiency (EE), i.e., the mass proportion of VCO encapsulated versus the total amount of oil used to synthesise the capsules, was determined as proposed by Concha et al. [19].

The thermal stability of the capsules was evaluated through thermogravimetric analysis (TGA) using a Netzsch STA 409 PC (Wittelsbacherstraße, Germany) equipment configured in a range of temperatures between 20–600 °C, and a constant N_2_ flow of 50 mL/min ensuring an inert atmosphere. The mechanical stability of the capsules was evaluated via compressive mechanical tests using a universal testing machine, Zwick/Roell Z005 (Ulm, Germany), with a load cell of 1kN and a load speed of 0.2 mm/min. Capsules were tested at two preconditioning temperatures: 20 °C and 160 °C.

### 2.7. Manufacture and Characterisation of Asphalt Mixtures Incorporating Capsules

Marshall specimens with 10 cm diameter and 6 cm height were manufactured in this study. (1) First, the aggregates were heated at 160 °C for 24 h, while the bitumen was heated at 160 °C for 2 h. (2) Second, the aggregates and bitumen, added in a content of 5% wt. of mass of aggregates, were mechanically mixed at 100 rpm in a metallic bowl at 160 °C for 3.5 min. Once the aggregates were covered by the bitumen, the 1:1, 1:5, and 1:9 VCO capsules at 20 °C were added to the asphalt mixture in a content of 0.5% wt. of asphalt mixture being continuously mixed for 1 min. This content of capsules was used based on the recommendation by Norambuena-Contreras [31], concluding that this was an optimal content based on the evaluation of the physical, mechanical, and self-healing properties of dense asphalt mixtures. (3) Third, the mixture with capsules was poured and compacted into a Marshall mould previously heated, applying 75 blows on each side of the specimen. Afterwards, the Marshall specimens were cooled at ambient temperature for 24 h and extracted from the mould. A total of 20 specimens were manufactured for this study: 3 Marshall specimens per each VCO capsule design and 5 reference specimens without capsules. Finally, specimens were characterised by measuring the bulk density and air void content according to ASTM D-2726-00 [44] and ASTM 3203-05 [45], respectively.

## 3. Results and Discussion

### 3.1. Chemical Properties of the Bitumen Blends with and without VCO

**Figure 4:**
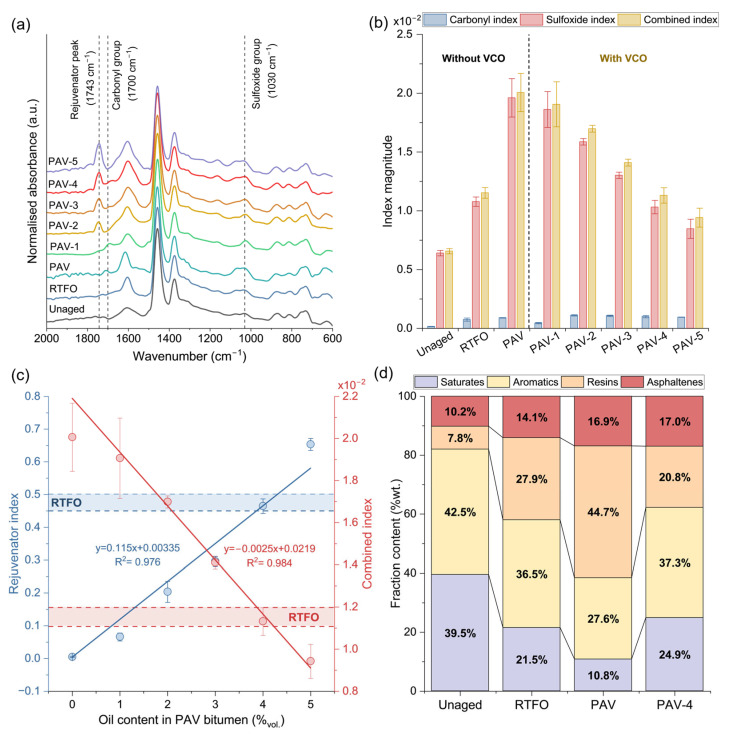
(**a**) FTIR-ATR curves and (**b**) average results of carbonyl, sulfoxide, and combined ageing indices for the unaged, RTFO, PAV, and PAV-1 to five bitumen samples; (**c**) average results of rejuvenator and combined ageing indices for PAV and PAV-1 to five fitted to a linear regression; (**d**) SARA fractions for the unaged, RTFO, PAV, and PAV-4 bitumen samples.

Figure 4a shows the representative FTIR-ATR curves for the unaged, RTFO, and PAV bitumen, with and without VCO addition. Particularly, chemical changes in the sulfoxide (1030 cm^−1^) and carbonyl (1700 cm^−1^) functional groups have been proposed by many researchers as the main indicators of the oxidation of a virgin bitumen. In this sense, Figure 4a shows an increase in the carbonyl and sulfoxide functional groups when the unaged bitumen was subjected to the successive RTFO and PAV ageing processes, with a more visible increase in the sulfoxide functional group when compared to the carbonyl one. To estimate such changes, Figure 4b shows the average results of the $C_{I}$ $\mathrm{and}\;S_{I}$ indices as well as the combined ageing index describing the overall oxidation process in the bitumen. From this Figure, the average combined index increased from 0.656 × 10^−2^ (SD: 0.22 × 10^−3^) to 2 × 10^−2^ (SD: 1.62 × 10^−3^) when the unaged bitumen was oxidised to a PAV state. Such variations were mainly attributed to changes in the sulfoxide functional group than in the carbonyl one and explained by the greater chemical reactivity of the initial sulfur content in the virgin bitumen with oxygen during the ageing process [19].

When the VCO is added as a rejuvenator into a PAV bitumen, Figure 4a shows that both carbonyl and sulfoxide indices decrease. Moreover, a characteristic peak at 1743 cm^−1^ is identified proportionally increasing with the VCO content in the PAV bitumen. This peak is attributed to the presence of carbonyl groups (C=O) of the VCO, as previously characterised by Concha et al. [19]. In fact, Figure 4b shows that, once the VCO is added to the PAV-aged bitumen, the combined index proportionally decreased following a linear tendency. Given the purpose to rejuvenate a PAV bitumen up to a RTFO condition, it is seen that the addition of 1% vol. VCO had no significant effect on the reduction of the combined index when compared to the PAV one. Nonetheless, the addition of 4% vol. VCO into the PAV bitumen decreased the combined index to values close to the RTFO condition. With this result, it is hypothesised that the addition of 4% VCO should be an appropriate content to rejuvenate the PAV oxidised bitumen up to the RTFO condition.

A better understanding of the reduction in the ageing index with the addition of VCO in a PAV bitumen can be seen in Figure 4c, showing the average results of the rejuvenator index $R_{I}$ (blue curve) and the combined index (red curve) as a function of the VCO content in the PAV bitumen. This Figure shows that the increase in the $R_{I}$ and the decrease in the combined ageing index are described as linear relationships as showing their respective fitting equations. With these equations, the previously hypothesised rejuvenating effect of the addition of 4% of VCO into the PAV bitumen can be verified as follows. Firstly, Figure 4c shows the variation of the combined index for the RTFO bitumen (the rejuvenation target) represented by the red interval “RTFO”. Secondly, by replacing this range in the combined index equation (red curve), the associated theoretical VCO content can be stated in a range of 3.96% to 4.32% vol. which encloses the PAV-4 bitumen sample. This is also verified by replacing the previous contents into the VCO rejuvenator index equation (blue curve) obtaining *R_I_* values varying between 0.45 and 0.50 (blue interval “RTFO”), which also enclose the average VCO rejuvenating index associated to the PAV-4 sample.

The previous findings using the FTIR-ATR technique can be contrasted by measuring the effect of the addition of 4% VCO in the SARA fractions of a PAV-aged bitumen. Figure 4d shows the results of SARA fractions for the unaged, RTFO, PAV, and PAV-4 bitumen samples. This Figure shows that the saturates and aromatics were reduced in −45.6% and −14.1% from an unaged to RTFO state, and −49.8% and −24.4% from a RTFO to PAV state, respectively. On the contrary, the resins and asphaltene fractions increased in +257.7% and +38.2% from unaged to RTFO state, and +60.2% and +19.9% from the RTFO to the PAV state, respectively. Overall, major changes were produced during primary ageing condition, with a significative increase in the resins mainly explained by the generation of resins from aromatics associated with the oxidative process at high temperature.

As an indicator of the overall oxidation process from the unaged to PAV ageing state, the asphaltene-to-maltene ratio increased from 0.114 to 0.203, so the bitumen increases its stiffness becoming prone to cracking. When incorporated the 4% vol. of VCO, the asphaltene-to-maltene ratio is similar to the PAV-aged condition (0.204). Nonetheless, when analysed the fractions associated with the maltene fraction, significative changes are noted. Particularly, the PAV-4 bitumen sample increased the saturates and aromatics in +130.56% and +35.15%, respectively, and reduced the resins in −53.47% with respect to the PAV-aged bitumen. The variations in the maltenic components for the PAV-4 resulted in the saturates and aromatics fractions being similar to the RTFO bitumen, with the resins content being even lower than the RTFO-aged sample. Since the asphaltenes fractions of the PAV-aged and the rejuvenated PAV-4 samples remained constant, it can be concluded that the VCO had a peptising effect, contributing to disperse the high-polar asphaltenes fraction of the PAV bitumen, resulting in a softening effect.

Finally, considering the effect of the oil evaluated via FTIR-ATR and SARA analysis, it can be concluded that the incorporation of VCO in a content of 4% vol. of PAV-aged bitumen restored the maltenic composition up to levels close to a RTFO aged condition.

### 3.2. Effect of the Oil Content on the Physical Properties of a PAV-Aged Bitumen

**Figure 5:**
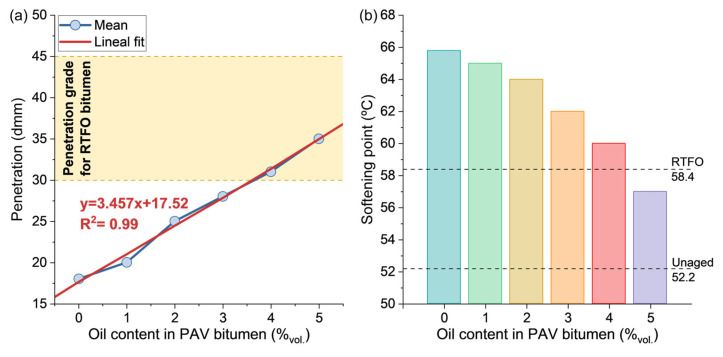
Results of (**a**) penetration and (**b**) softening point for PAV and PAV-1 to 5 bitumen samples.

Figure 5a shows that the penetration value increased linearly when the VCO content in the PAV-aged bitumen is augmented, as described by the linear fitting equation. A similar tendency was previously detected by Li et al. [46]. Based on the rejuvenation purpose, the addition of VCO should be able to restore the penetration values of a PAV bitumen up to a RTFO aged state (30–45 dmm) (see the colored area in the Figure). This condition is reached for a theoretical VCO content between 3.61% and 7.94%. It can be noticed that the minimum VCO content obtained here is in line with the value obtained under the FTIR-ATR analysis (see Section 3.1). This confirms that the appropriate amount of VCO to rejuvenate a PAV-aged bitumen should be closer to the lower limit obtained here, i.e., 4% addition. Based on these results, it is hypothesised that the addition of VCO into a PAV bitumen in concentrations closer to the theoretical maximum value (7.94% vol. bitumen) could negatively affect the performance of the pavement by the occurrence of permanent deformations such as rutting.

Additionally, Figure 5b shows the results of the softening point for each of the PAV-aged samples with different contents of VCO. As reference, this Figure shows the softening point values of the unaged (52.2 °C) and RTFO (58.4 °C) bitumen samples. The softening point decreased with the addition of the VCO content in the PAV-aged bitumen, from 65.8 °C to 57.0 °C, for the PAV-0 and PAV-5 bitumen samples, respectively, indicating a more ductile behaviour. Particularly, the softening point for the PAV-4 (60 °C) is closer to the softening point of the RTFO bitumen sample (58.4 °C). Since the softening point for the 4% and 5% VCO are above and below the RTFO reference value, this test suggests that the VCO content required for rejuvenation to a RTFO state may fall between these contents, reducing the maximum theoretical limit established by penetration test.

**Figure 6:**
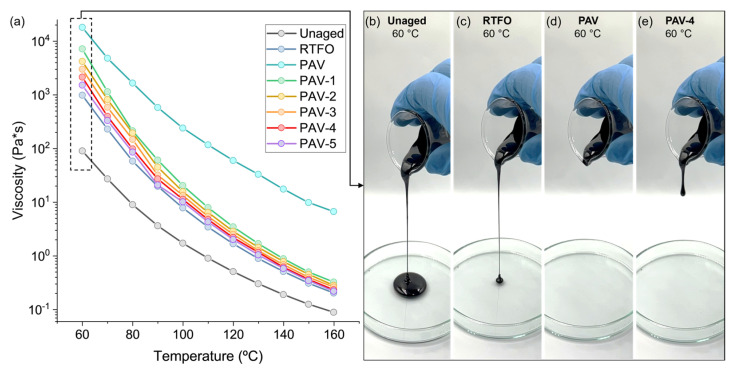
(**a**) Viscosity curves for virgin, RTFO, PAV, and PAV-1 to five bitumen samples, (**b**–**e**) examples images of unaged, RTFO, PAV, and PAV-4 bitumen samples flowing at 60 °C after 5 s.

Figure 6a shows the viscosity curves depending on the temperature, ageing process, and VCO content to rejuvenate the PAV-aged bitumen. Overall, these factors are seen: (i) a progressive reduction in the viscosity when the temperature is increased, (ii) an increase in the viscosity at each ageing process (RTFO and PAV), and (iii) a reduction of the viscosity for the PAV-aged sample with the incorporation of VCO. As an example, Figure 6b–e show images of the flow of the unaged, RTFO, PAV-4, and PAV bitumen samples at 60 °C after 5 s. Based on rejuvenation purpose, the addition of VCO to a PAV bitumen should be enough to decrease its viscosity up to a RTFO condition, facilitating its flow to seal a microcrack. In this sense, Figure 6a shows that the reduction in the viscosity for the PAV-VCO samples was a combined effect of the increase in the VCO and the temperature. Particularly, the addition of VCO contents between 4% and 5% significantly reduced the viscosity of the PAV-aged bitumen to values closer to the RTFO curve for most of the temperature range. Consequently, it can be concluded that adding VCO contents around 4–5% in a PAV bitumen should reduce this viscosity up to the RTFO state.

### 3.3. Effect of the Oil Content on the Rheological Properties of a PAV-Aged Bitumen

**Figure 7:**
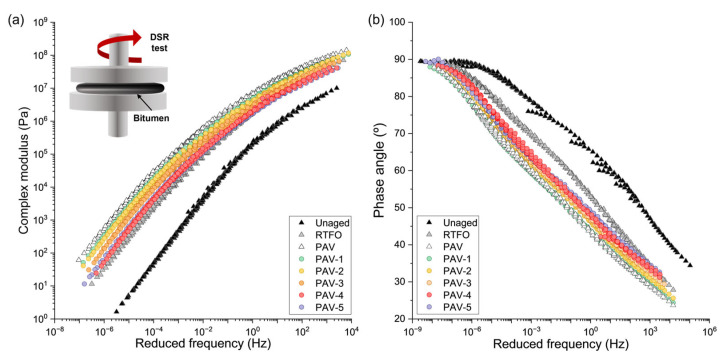
Master curves of (**a**) complex modulus (|G*|) and (**b**) phase angle (δ) for the unaged, RTFO, PAV, and PAV-1 to five bitumen samples at a reference temperature of 25 °C.

Figure 7 shows the master curves of the complex modulus |G*| and phase angle δ for the virgin, RTFO, and PAV bitumen samples with and without VCO at a reference temperature of 25 °C. Figure 7a shows an increase in |G*| for the virgin bitumen after the RTFO and PAV ageing processes, respectively. This phenomenon is mainly explained by the hardening of the virgin bitumen after each of the ageing processes, as previously confirmed by the increasing of the asphaltene-to-maltene ratio (see Section 3.1).

The hardening of the virgin bitumen after the RTFO and PAV ageing processes is also evidenced in Figure 7b. Overall, samples with δ closer to 90° indicate a more viscous behaviour such as that of a Newtonian viscous liquid, while δ closer to 0° indicate a more elastic behaviour such as that of an elastic solid [42]. Because of the increase in |G*|for the virgin bitumen after the RTFO and PAV oxidation processes, a reduction is expected of the δ for the virgin bitumen turning into a more elastic behaviour for all the range of frequencies evaluated. In Figure 7b, the hardening process from a virgin to a PAV-aged bitumen is visible, for example, at low frequencies. Here, the virgin bitumen developed a plateau zone with δ values closer to 90° for reduced frequencies up to 1 × 10^−6^ Hz. After the RTFO and PAV ageing processes, the plateau zone was proportionally reduced, indicating a more elastic behaviour.

As evidenced in Section 3.1 and Section 3.2, the addition of VCO in contents around 4% by vol. bitumen can restore the maltene fractions and the physical properties of the PAV-aged bitumen to values up to the RTFO-aged bitumen. Consequently, the rejuvenating effect of the VCO should also modify the rheological response of the PAV-aged bitumen. Figure 7a shows that the addition of VCO progressively reduced the |G*| of the PAV-aged bitumen for all the range of frequencies. This means that the PAV-aged bitumen decreased its stiffness when the VCO content is increased. The more significant reductions in |G*| happen at high frequencies (low temperatures), where the increase in the VCO contents resulted in |G*| values closer to the RTFO master curve. The PAV-4 and PAV-5 master curves are slightly below that of the RTFO for high frequencies (low temperatures), moderately above that of the RTFO for low frequencies (high temperatures), and overlap with the RTFO curve at intermediate frequencies (temperatures). Thus, adding such VCO contents restores this rheological property of a PAV-aged bitumen.

Similarly, Figure 7b shows that the addition of VCO to a PAV-aged bitumen increased the δ for all the range of frequencies indicating a more viscous behaviour. Particularly, the plateau zone previously described for the unaged bitumen at low frequencies is slightly extended with the VCO addition into the PAV-aged bitumen, meaning that the viscous behaviour of the PAV rejuvenated bitumen can stand for a wider time range (temperatures range). Particularly, the PAV-4 and PAV-5 samples overlap with the RTFO curve at the lowest frequencies (high temperatures) and highest frequencies (low temperatures), meaning that the rejuvenation effect of the PAV bitumen is more effective under such conditions than at intermediate temperatures. Considering the similar rheological behaviour for the PAV-4 and PAV-5 samples with the RTFO-aged bitumen, the master curves of |G*| and δ suggest that the addition of VCO in a content of 4% by vol. of bitumen restore the rheological response of a PAV-aged bitumen to levels similar to an RTFO state. Finally, based on the chemical (Section 3.1), physical (Section 3.2), and rheological (Section 3.3) properties, it can be concluded an appropriate dose of VCO to rejuvenate a PAV-aged bitumen up to a RTFO state ranging between 3.61% (given by penetration test and 4.00% vol. of bitumen (given by FTIR-ATR analysis and rheological properties). In the next sections, an equivalent dosification of VCO capsules will be determined.

### 3.4. Effect of the Oil Content on the Physical Stability of the Capsule’s Components

**Figure 8:**
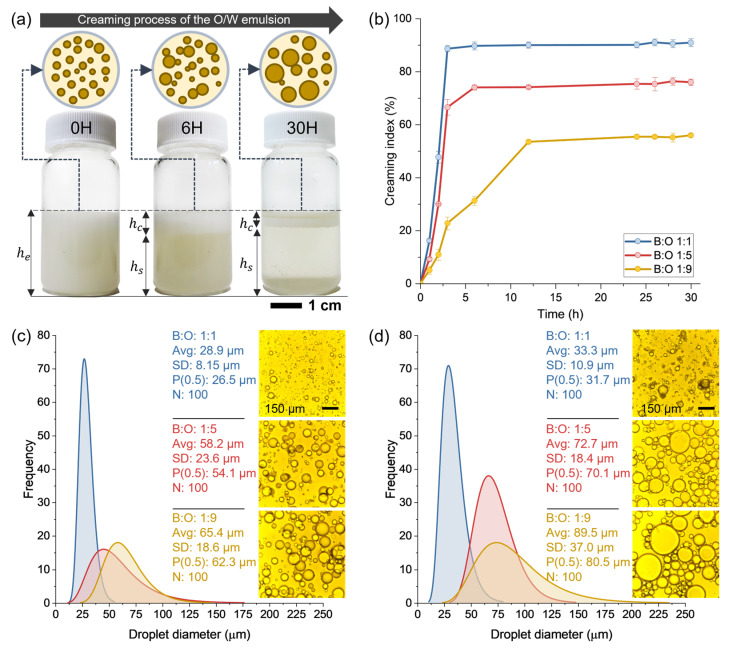
(**a**) Creaming phenomenon occurring in the O/W emulsion over time; (**b**) average results of creaming index for O/W emulsion with B:O ratios of 1:1, 1:5, and 1:9; and VCO droplet diameter distribution in the O/W emulsion fitted to a log-normal distribution at (**c**) 1 h and (**d**) 30 h.

During the encapsulation process, the O/W emulsion should be physically stable to ensure the proper synthesis of the capsules, i.e., no phase separation (creaming) should occur during the encapsulation. Figure 8a exemplifies the creaming instability occurring in an O/W emulsion and Figure 8b shows the average results of the creaming index for the O/W emulsions based on the B:O ratios 1:1, 1:5, and 1:9. From this Figure it is seen that, overall, the creaming phenomenon of the O/W emulsions was highly dependent of the time, progressively increasing up to a constant quasi-stationary state.

Figure 8b also shows that the B:O ratio highly influenced the way each O/W emulsion was creamed over time. Particularly, the more the VCO content, the lower the creaming index was. In addition, the creaming indices over time were described in different stages. The emulsion based on B:O ratio 1:1 presented: (i) a linear phase after 3 h with creaming index values up to 88.7% and, then, (ii) a quasi-stationary stage was observed, maintaining the creaming index at a constant value of around 90%. The emulsions with B:O ratios of 1:5 and 1:9 presented: (i) a linear stage after 3 h with creaming indices of 66.55% and 22.74%, respectively; (ii) a transition phase between 3–6 h with a creaming index of 74.05% for the 1:5 emulsion and between 3–12 h with a creaming index of 53.5% for the 1:9 emulsion; and (iii) a quasi-stationary phase between 6–30 h with a creaming index of 76.03% for the 1:5 emulsion and between 12–30 h with a creaming index of 55.9% for the 1:9 emulsion. Based on these results, it is seen that the transition stage was kept for a longer time when the VCO proportion increased in the B:O ratio from 1:1 to 1:9.

Consequently, it is concluded that the increase in the VCO content in the O/W emulsion improved its physical stability, retarding the separation of the oil from the alginate solution. As previously described by Concha et al. [19], creaming phenomenon is closely related to the coalescence of the oil droplets with time. As a result of this, larger droplets can ascend easier to the top of the emulsion promoting the phase separation between the VCO and the alginate solution. So, the key factor to reduce the coalescence phenomenon is the reduction of the movement of the larger oil droplets, so the creaming process is retarded. This can be achieved by increasing the viscosity of the emulsion.

To probe the effect of the viscosity in the size of the oil droplets with time, Figure 8c,d show representative fluorescence microscopy images and their respective size frequency distributions for each O/W emulsion at 1 h and 30 h of response, respectively. From this Figure it is seen that larger droplets are obtained when decreased the B:O ratio and with time. The representative VCO droplet diameter for the emulsions with B:O ratios 1:1, 1:5, and 1:9 was 26.5 µm, 54.1 µm, and 62.3 µm at 1 h, and 31.7 µm, 70.1 µm, and 80.5 µm at 30 h, respectively. With these results, it should be expected that the emulsion with a high VCO content promote coalescence of their larger oil droplets accelerating the creaming process. Nonetheless, as reported in Section 2.6, the emulsion with B:O ratio of 1:9 presented the highest viscosity retarding the ascension of the larger droplets, improving its physical stability. Finally, based on the previous results, it is concluded that the creaming phenomenon of the O/W emulsions was highly dependent on the B:O ratio. As consequence, O/W emulsions with a higher VCO content can be potentially used for a longer period during the encapsulation process. Their physical stability helps to provide more time for appropriate encapsulation of the emulsion. These results also show the importance of starting the encapsulation process right after synthesising the O/W emulsion.

### 3.5. Effect of the Oil Content on the Physical, Thermal, and Mechanical Performance of the Capsules

**Figure 9:**
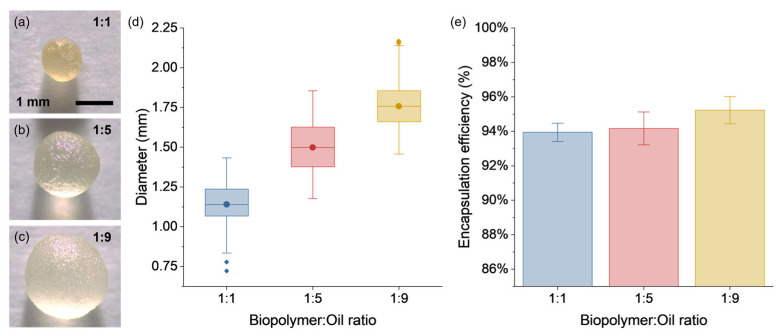
Optic images for (**a**) 1:1, (**b**) 1:5, and (**c**) 1:9 VCO capsules; (**d**) box-plot graphs of the diameter; and (**e**) average results of the encapsulation efficiency for the 1:1, 1:5, and 1:9 VCO capsules.

Figure 9a–c show VCO capsules with regular spherical morphology for B:O ratios of 1:1, 1:5, and 1:9, which exhibited sphericity factors of 0.049, 0.047, and 0.036, respectively. According to this sphericity criterion, factors under 0.05 indicate capsules with regular spherical morphology, while values close to the unit represent capsules with elongated morphology [19]. Figure 9d shows box plots of the size of each capsule design. From this Figure, the B:O ratios of 1:1, 1:5, and 1:9 resulted in capsule sizes with average values of 1.14 mm (SD: 0.13 mm), 1.49 mm (SD: 0.16 mm), and 1.76 mm (SD: 0.16 mm), respectively. In this study, the concentration of alginate in the aqueous solution and the nozzle diameter of the encapsulator were kept constant, so the increase in the size of the capsules is attributed to the larger VCO droplets being encapsulated within the alginate matrix, increasing the total amount of the VCO. Increasing the VCO content should result in capsules with thinner alginate-wall cavities, as shown previously by Xu et al. [47].

Figure 9e shows the average results of the encapsulation efficiency for all the B:O ratios. Overall, it is seen that in all cases a high encapsulation efficiency was obtained with averages values of 93.94% (SD: 0.5%), 94.20 (SD: 1.0%), and 95.23% (SD: 0.8%) for the 1:1, 1:5, and 1:9 B:O ratios, respectively. Capsules with higher VCO content retained a slightly greater amount of VCO per gram of capsule inside its polynuclear internal structure.

Based on the method used to determine the encapsulation efficiency, the amount of encapsulated VCO per gram of capsules with B:O ratios of 1:1, 1:5, and 1:9 was 0.217 g, 0.422 g, and 0.601 g, respectively. These large differences in the mass of VCO will significantly influence the capsule dosage when used to rejuvenate an aged bitumen.

The equivalent dosage of capsules per total weight of bituminous material, whether it is bitumen, mastic, or asphalt mixture, can be determined using Equation (5):

(5) $$Capsule\;content\;\left(\%\;wt.\right)=\frac{W_{b}\times {\%}_{oil}\times W_{cap}\times \rho_{oil}}{W_{eo}\times W_{bm}\times \rho_{b}}\times 100$$

where $W_{b}$ is the weight of the bitumen, in g; $\rho_{b}$ is the density of the bitumen, in g/cm^3^; ${\%}_{oil}$ is the percentage of oil per volume of bitumen required for rejuvenation; $W_{cap}$ is the mass of capsules to calculate the encapsulation efficiency, in g; $W_{eo}$ is the mass of the encapsulated oil, in g; $\rho_{oil}$ is the density of the oil, in g/cm^3^; and $W_{bm}$ is the mass of the bituminous material (bitumen, mastic, or asphalt mixture), in g. If the capsule content is determined for dosage in bitumen, $W_{bm}$ is equal to $W_{b}$.

**Figure 10:**
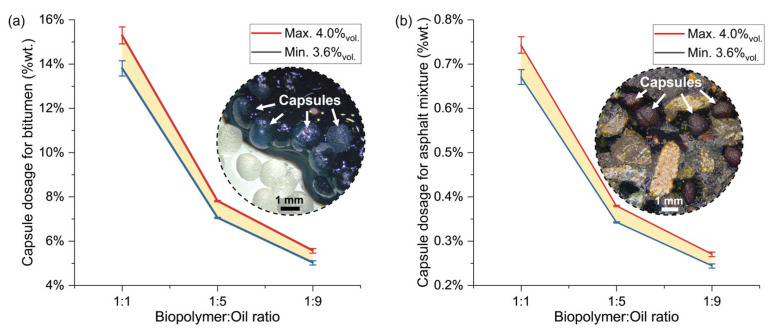
Minimum and maximum dosage curves of VCO capsules with different biopolymer:oil ratios inside the (**a**) bitumen and (**b**) dense asphalt mixture.

Figure 10a,b show the theoretical capsule dosages required to rejuvenate PAV-aged bitumen up to the RTFO state. The results from the previous sections indicated that the ${\%}_{oil}$ required for this effect was between 3.6% and 4.0% vol. Since the weight of the bitumen is approximately 5% of the weight of the asphalt mixture, when dosing capsules as a percentage of the asphalt mixture weight, the percentage of capsules for asphalt mixtures 20 times lower than for the bitumen. Interestingly, the dosage magnitudes of capsules in both bituminous materials are in line with the capsule contents used in previous research [31]. It is important to mention that the capsule dosages presented here were calculated assuming full release of the VCO and complete diffusion of VCO in the binder.

**Figure 11:**
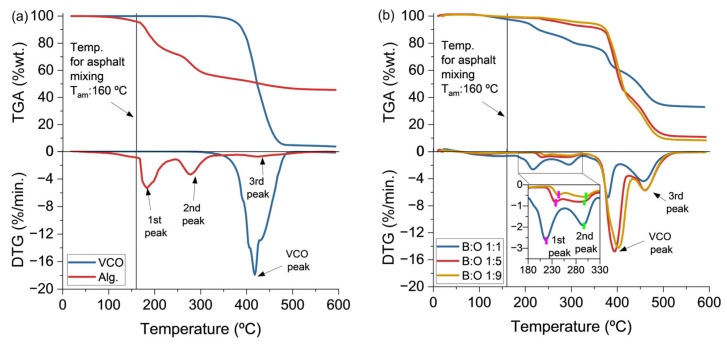
TGA–DTG curves for (**a**) VCO and alginate; and (**b**) capsules with B:O ratios 1:1, 1:5, and 1:9.

Otherwise, Figure 11a shows that the alginate biopolymer is characterised by a three-step thermal degradation consisting of (i) a first step at 185 °C, as indicated by the DTG curve, associated to the loss of initial moisture and the chemical dehydration of the biopolymer; (ii) a second degradation step was recorded at 278 °C which was associated with the fracture of glycosidic bonds, decarboxylation and decarbonylation, releasing H_2_O, CO_2_, and other light compounds; and (iii) a third degradation step recorded at 417 °C attributed to a further degradation of residues [48]. On the contrary, VCO degrades at a single step with a maximum degradation occurring at 417 °C as indicated by its respective DTG curve. This loss of mass was attributed to the decomposition of volatiles associated to polyunsaturated (linoleic acid) and monounsaturated (oleic acid) fatty acids [49].

Additionally, Figure 11b shows the TGA–DTG curves for the capsule designs depending on the B:O ratio. From this Figure, it is evidenced that the addition of the VCO in the capsules increased their thermal stability. This can be appreciated along the DTG curve of each capsule design as follows. First, the three degradation peaks of the alginate were proportionally shifted with the addition of VCO. Accordingly, capsules with B:O ratio 1:1 started to degrade earlier than capsules with 1:5 and 1:9 ratios (see the first, second, and third alginate peaks in Figure 11b. Second, the characteristic VCO degradation peak appeared early for the capsules in comparison with the pure oil. Nonetheless, the VCO peak was slightly shifted to a higher temperature with the addition of VCO into the capsule. Finally, in reference to the temperature of asphalt mixing (T_am_), around 160 °C, the capsules with B:O 1:1 presented a loss of mass close to 5%, while those with a B:O ratio 1:5 and 1:9 presented no thermal degradation. Based on the previous analysis, it is concluded that capsule designs based on B:O 1:5 or 1:9 could be appropriate to be potentially incorporated into asphalt mixtures for asphalt self-healing purposes.

**Figure 12:**
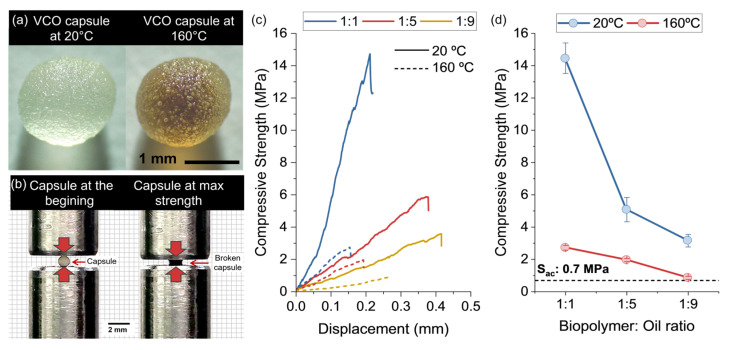
(**a**) Optic images of VCO capsule with B:O ratio 1:5 at 20 °C and 160 °C; (**b**) representation of the compression test performed in the VCO capsules; (**c**) representative compression curves; and (**d**) average results of the compression strength for each VCO capsule at 20 °C and 160 °C.

Figure 12a shows the morphological effect that the thermal conditioning has on a capsule with B:O ratio 1:5. This image is evidence that all capsules experienced a reduction in their sizes after the thermal conditioning. To evaluate if this size-reduction effect has an impact on the mechanical performance of the capsules, they were subjected to compression tests (see Figure 12b–d). Figure 12c shows the representative compression curves for each capsule design at 20 °C and 160 °C and Figure 12d shows the average results of the mechanical compressive strength for capsules conditioned at 20 °C and 160 °C. These figures show that the compressive strength of the capsules decreased with the increment of the B:O ratio presenting a more ductile behaviour, and that capsules conditioned at 160 °C significantly reduced their compressive strength when compared to those at 20 °C.

Possible reasons explaining the reduction of the compressive strength by the combined effect of the B:O ratios and the conditioning temperature can be: (i) the weakening of the capsule by thinning of the alginate-wall cavities when increasing the B:O ratio, and (ii) the mass loss of the alginate at T_am_ = 160 °C, as was previously seen by TGA tests. Despite these drawbacks, the compressive strength of the capsules was still higher than the maximum reference pressure for compaction of an asphalt mixture (S_ac_: 0.7 MPa), as reported by Delgadillo and Bahia [50]. Particularly, capsules showing compressive strength values higher than S_ac_ could cause their activation to be difficult, while capsules with compressive strength values closer to S_ac_ could be potentially damaged during the asphalt compacting. Thus, it is concluded that a capsule design with a B:O ratio 1:5 could be the most appropriate design for its incorporation in asphalt mixtures for asphalt self-healing purposes.

**Figure 13:**
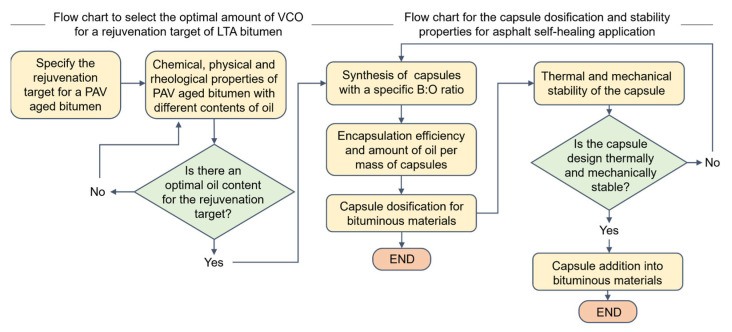
Flowchart showing the process to select the optimal amount of rejuvenator and the equivalent dosification in terms of capsules for a given rejuvenation target and the process to determine the equivalent dosage of VCO capsules and their stability properties for asphalt self-healing.

Considering the (i) chemical, physical, and rheological characterisations of PAV-aged bitumen with VCO addition, and (ii) the physical properties, and thermal and mechanical analysis of the VCO capsules, a step-by-step procedure to select the optimal amount of rejuvenator and the equivalent dosage in terms of capsules, for a given rejuvenation target, is presented in Figure 13 in a flowchart format.

### 3.6. Effect of the Capsule Design on the Physical Properties of Asphalt Mixtures

**Figure 14:**
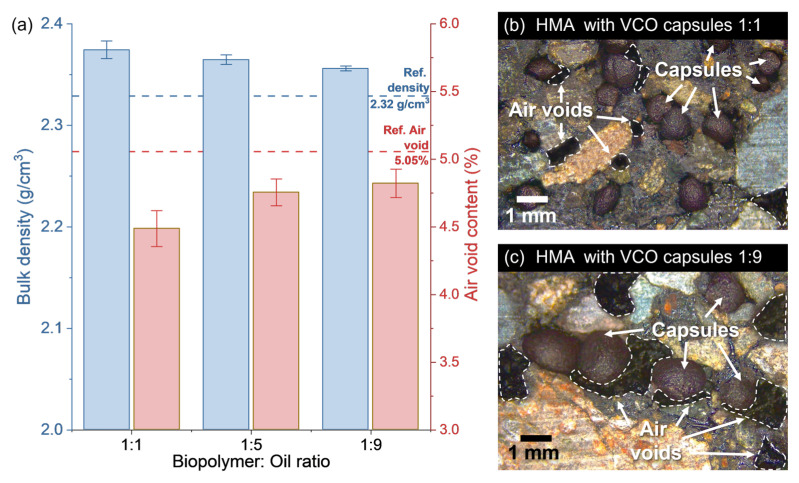
(**a**) Average results of bulk density and air void content for asphalt mixtures samples with addition of capsules with B:O ratios of 1:1, 1:5, and 1:9; and optic microscopy images of hot-mix asphalt (HMA) incorporating capsules with B:O ratios of (**b**) 1:1 and (**c**) 1:9.

Figure 14a shows the average results of bulk density and air void content for the semidense hot-mix asphalt with and without the addition of capsules (i.e., bitumen content of 5% wt. of aggregates and capsule content of 0.5% wt. of asphalt mixture). From this Figure it is seen that the asphalt mixtures incorporating capsules increased the bulk density when compared to a reference asphalt mixture without capsules with a value of 2.32 g/cm^3^. Moreover, the mixtures with VCO capsules decreasing their B:O ratio from 1:1 to 1:9 significantly decreased the bulk density of the asphalt mixture from 2.37 g/cm^3^ (SD: 0.0087 g/cm^3^) to 2.35 g/cm^3^ (SD: 0.0024 g/cm^3^), respectively. Overall, such changes detected in the bulk density can be attributed to variations of the total volume and mass of the mixture with the addition of capsules. Nonetheless, since capsules were added into the mixture in a constant value of 0.5% wt. of asphalt mixture, changes in the bulk density are mainly attributed to variations in the total volume of the mixture. Similar results have been previously reported by Norambuena-Contreras et al. [31].

To probe it, Figure 14a also shows that the air void content for the mixtures with capsule addition was reduced in comparison with a reference asphalt mixture, with a value of 5.05%. From this, it is seen that capsules acted as a filler additive inside the asphalt mastic affecting the volumetric properties of the asphalt mixture. Particularly, when the B:O ratio of the VCO capsules decreases from 1:1 to 1:9, the air void content of the mixture is significantly increased from 4.48% (SD: 0.133%) to 4.82% (SD: 0.105%), respectively. As an example of the increase in air voids, Figure 14b,c show some images of asphalt mixture incorporating VCO capsules with B:O ratios of 1:1 and 1:9, respectively.

A reason explaining this tendency is directly related to the size of the capsules previously discussed in Section 3.5. In this manner, for a given volume of air void, smaller capsules design with high specific surface can more effectively fill the total void volume in comparison to those capsules with higher sizes and lower specific surface. It is also hypothesised that the superficial VCO content in the capsules as well as the partial release of the encapsulated VCO during mixing and compaction of the asphalt mixture act as lubricant contributing to better compact the mixture, increasing the bulk density. From the previous analysis, it is concluded that the addition of VCO capsules had a significant effect on the bulk density, where such variations were highly dependent on the capsule morphology, so the lower the size of capsule, the lower the air void content and the higher the bulk density of the asphalt mixture.

## 4. Conclusions

This paper evaluated the effect of the design parameters of encapsulated rejuvenators on their physical, thermal, and mechanical properties for asphalt self-healing purposes. The main results showed that the evaluation of the chemical (FTIR, and SARA), physical (penetration, softening point, and viscosity), and rheological (|G*| and δ) properties in the LTA bitumen contributed to determine a VCO content of 4% by vol. to rejuvenate a PAV-aged bitumen up to a RTFO ageing state (rejuvenation target). Moreover, assuming total liberation of the VCO from capsules and complete diffusion through the binder, the equivalent capsule dosage for the rejuvenation target was determined. It varied between 5.03% wt. to 15.3% wt. of the bitumen, or 0.24% wt. to 0.74% wt. of the asphalt mixtures, depending on the B:O ratio of the capsule. Based on the results, the following main conclusions were drawn:

- Chemical analysis by FTIR-ATR proved that adding a 4% vol. of VCO into a PAV-aged bitumen reduced its ageing indices to a level similar to the RTFO ageing state. For this VCO content, SARA analysis showed a softening effect for the PAV-aged bitumen, significantly increasing the saturates (+130.56%) and aromatics (+35.15%);
- Physical characterisation showed that a 4% vol. of VCO into a PAV-aged bitumen increased the penetration and decreased the softening point and viscosity to the RTFO condition. From these tests, an optimal minimum VCO content was stated at 3.61% vol. bitumen for the rejuvenation purpose;
- Rheological analysis evidenced a reduction in |G*| and an increase in δ with the addition of VCO into a PAV bitumen, resulting in a softer and more viscous bitumen. A VCO content of 4% vol. bitumen was the recommended dosage to recover the rheological properties of a PAV-aged bitumen to the RTFO state;
- Creaming tests revealed that increasing the B:O ratio from 1:1 to 1:9 improved the physical stability of the O/W emulsions for synthesising VCO capsule. Capsule synthesis should be conducted promptly after emulsion synthesis to prevent instability;
- Reducing the B:O ratio from 1:1 to 1:9 produced larger capsules (1.14 mm to 1.76 mm), with high encapsulation efficiencies (93.94% to 95.23%) and high VCO content per gram of capsules (0.217 g to 0.601 g). For the rejuvenation target, capsules with high B:O ratio required a higher dosage than those with low B:O ratio;
- VCO capsules with low B:O ratios presented better thermal stability but lower mechanical stability. Capsules with a B:O ratio of 1:5 presented no thermal degradation and a compression strength over the compaction of an asphalt mixture;
- Bulk density variations in the mixtures with the addition of VCO capsules depended on the morphology of the capsules, so the lower their size, the lower the air void content and the higher the bulk density of the asphalt mixture.

Finally, from the conclusions drawn in this study, the authors propose future work on the synthesis of capsules focused on: (i) the optimisation of the capsule design under a factorial approach varying both the B:O ratio and the hardening solution concentration, and (ii) quantifying the effect of the content of the optimised capsule on the rheological, physical, mechanical, and self-healing properties of mastic and asphalt mixtures.

## Figures and Tables

**Table 1:** Particle size distribution of the aggregates used to manufacture dense asphalt mixture.

Sieve Size (mm)	% Passing
19	100
12.5	84
10	72
4.75	50
2.36	35
0.6	16
0.3	11
0.15	7
0.075	5

## Data Availability

The data that support the findings of this study are available on request from the corresponding author.
